# Electron Paramagnetic Resonance Spectroscopy Reveals Promoter Dependent Transcription Regulation by Copper Activated CueR in *Pseudomonas aeruginosa*

**DOI:** 10.1002/cphc.202500625

**Published:** 2026-01-25

**Authors:** Ameer Yasin, Misan Irshed, Lukas Hofmann, Yulia Shenberger, Lada Gevorkyan‐Airapetov, Sharon Ruthstein

**Affiliations:** ^1^ Department of Chemistry and Institute of Nanotechnology and Advanced Materials, Faculty of Exact Sceinces Bar‐Ilan University Ramat‐Gan Israel

**Keywords:** copper homeostasis, CueR, double electron electron resonance, DNA spin‐labeling, metalloregulator

## Abstract

Metal responsive transcription factors are essential for bacterial metal homeostasis, allowing cells to regulate metal uptake, efflux, and detoxification in response to fluctuating metal ion levels. Among these, CueR, a member of the MerR family, is widely found in Gram‐negative bacteria. While *E. coli* CueR has been extensively studied, revealing that it adopts multiple conformational states to regulate transcription, *P. aeruginosa* CueR (PACueR) remains less characterized, with no resolved structure despite regulating a broader set of genes. In this study, we applied electron paramagnetic resonance (EPR) spectroscopy combined with DNA spin‐labeling to investigate the conformational states of PACueR bound to two different promoter sequences, copZ2 and mexPQ‐opmE. We examined the effects of PACueR binding and copper addition, capturing the transcription initiation stage that represents an essential step in copper homeostasis regulation of *P. aeruginosa*. Our results reveal promoter‐specific differences in PACueR DNA interactions, suggesting that while the core transcription initiation mechanism is conserved, variations in promoter affinity and length of dyad symmetry fine‐tune transcription levels in response to copper. These findings highlight the value of EPR spectroscopy in probing metal‐dependent transcription mechanisms and offer new insights into copper regulation in *P. aeruginosa*, a clinically important pathogen.

## Graphical abstract

We performed electron paramagnetic resonance (EPR) experiments to reveal that *P.* *aerguinosa* CueR copper transcription factor enhances expression of copZ2 more strongly than mexPQ‐opmE under copper stress, consistent with their detoxification roles. Findings highlight EPR as a key tool for probing metal‐regulated transcription.

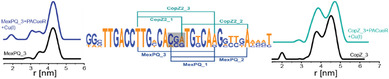

## 1 Introduction

Metal‐responsive transcription factors are essential regulators in bacteria, responsible for maintaining metal ion homeostasis and protecting cells from metal‐induced toxicity [1, 2, 3, 4]. These proteins sense the intracellular levels of critical transition metals such as zinc, copper, iron, and nickel, and accordingly regulate gene expression for metal uptake, efflux, storage, and detoxification [2, 5, 6]. They typically achieve this by binding directly to metal ions, which induces conformational changes that modulate their DNA‐binding activity. This regulation enables bacteria to swiftly adapt to fluctuating metal availability, an especially important feature during environmental changes or host infection [2, 5, 6, 7]. They can generally be classified as either transcription activators or repressors [2]. In both cases, metal ion binding influences the transcription of downstream genes involved in detoxification or export of the metal. However, the molecular mechanisms differ in activators, metal binding enhances DNA binding and induces bending of the DNA to promote transcription. In repressors, metal binding leads to dissociation from the DNA, thereby relieving repression. A well‐studied example of a metal‐activated transcription activator is *E. coli* CueR, a homodimer protein that responds to copper ions. CueR's structure has been resolved via X‐ray crystallography in various functional states: the apo form, the holo‐form (bound to Cu(I)), the DNA‐bound repressed state, and the active state bound to DNA and Ag(I), a Cu(I) mimic [8, 9]. Interestingly, while only minor structural changes are observed in CueR itself, a significant 36° DNA bend occurs upon activation, raising questions about how CueR facilitates transcription without substantial conformational rearrangement.

Investigating metal‐responsive transcription factors like CueR provides key insights into bacterial physiology and pathogenesis, as well as potential targets for antimicrobial intervention [10, 11, 12]. These systems exemplify precise protein‐ligand specificity and dynamic regulation. A combination of biophysical techniques, including X‐ray crystallography, fluorescence resonance energy transfer (FRET), and cryo‐electron microscopy (cryo‐EM), and electrophoretic mobility shift assays (EMSA), has been used to probe the structure, binding affinities, and thermodynamics of these complexes [8, 9, 13, 14, 15, 16, 17, 18, 19, 20]. However, each method has its own advantages and limitations, which leave gaps in understanding the transcription mechanism. Electron paramagnetic resonance (EPR) spectroscopy has become a useful tool to shed light on complex biological reaction mechanisms. Its main advantages are that it can provide dynamical and conformational information on any biological system, without the limitation of protein size or the requirement for crystallization [21, 22, 23, 24, 25, 26, 27].

Using EPR, we investigated the dynamic conformational states of *E. coli* CueR during transcriptional regulation [28, 29, 30, 31, 32, 33]. Our results reveal five distinct functional states: (1) apo (metal‐free), (2) holo (metal‐bound), (3) repressed (DNA‐bound), and two active forms: (4) a transcriptionally active state with one Cu(I) per monomer, and (5) a transcriptionally inactive “dead‐end” state with two Cu(I) ions per monomer. Notably, only the singly bound active form leads to transcription. Furthermore, through a newly developed DNA spin‐labeling method, we demonstrated that DNA bending can occur independently of metal ions, though Cu(I) facilitates this process, providing insights into the metal's role in transcription initiation [34].

**FIGURE 1:**
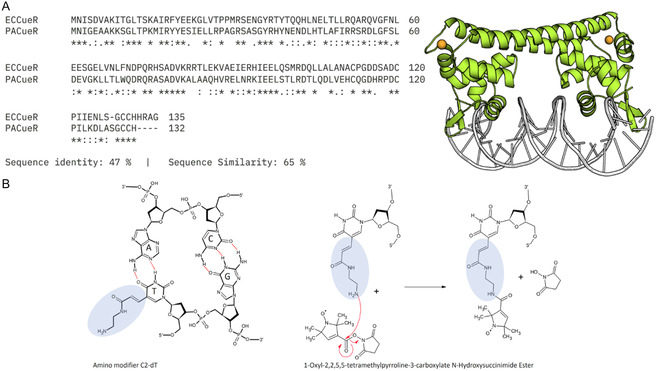
(A) CueR protein sequence alignment between *E. coli* and *P. aeruginosa* revealing a 47% identity and 65% similarity between the two species. PACueR homology model AlphaFold ID: Q9HV30, representing the structure of a classical MerR metalloregulator family member [35]. Orange spheres represent bound copper Cu(I), and the DNA sequence is shown in white tubing, while both monomers are depicted in green cartoon representation. (B) DNA spin labeling methodology. Thymine nucleotide with modified C2 linker. Subsequently, a 1‐oxyl‐2,2,5,5,‐tetramethylpyrroline‐3‐carboxylate N‐hydroxysuccinimide ester spin‐label is chemical attached [36].

Building on these findings, we extended our study to *P. aeruginosa* CueR (PACueR), which lacks a resolved crystal structure but shares ≈47% sequence similarity with *E. coli* CueR (Figure 1A) [3, 8, 37]. While *E. coli* CueR regulates two genes (copA and cueO), PACueR is regulating the transcription of at least five genes copZ1, copZ2, mexPQ‐opmE (PA3521−3523), copA1 (PA3920), and PA3515−3519 [18, 38, 39], highlighting its expanded regulatory role and potential contribution to the bacterium's pathogenicity. Recently, we applied continuous wave (CW) EPR spectroscopy to monitor changes in protein dynamics upon Cu(I) binding and interaction with two distinct DNA promoters: copZ2 and mexPQ‐opmE [40]. This study used spin‐labeling at the protein itself and showed differences in EPR spectra between these two promoter complexes, suggesting differential DNA‐protein interactions that may underlie promoter‐specific transcriptional outcomes.

In this work, we extend our investigation and focus specifically on the conformational changes in DNA induced by coordination PACueR and Cu(I), using a straightforward and robust nitroxide DNA spin‐labeling method developed in our lab (Figure 1B) [36]. This approach allows us to gain additional mechanistic insights into the metal‐induced transcriptional activation process in this clinically relevant pathogen.

## 2 Results

**FIGURE 2:**
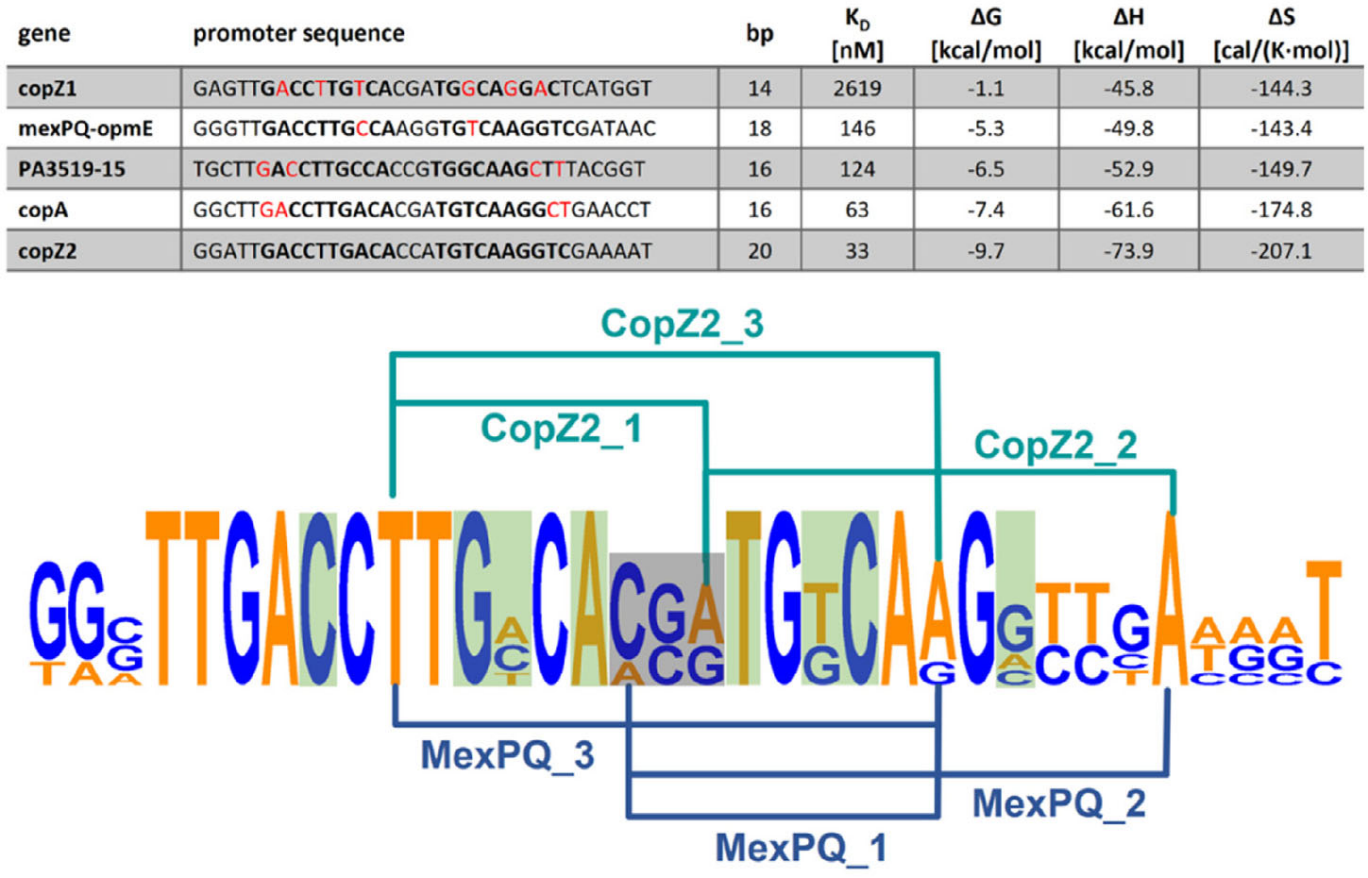
Five palindromic promoter sequences regulated by PACueR. The dyad symmetry is depicted in bold while mismatches are highlighted in red. The bp indicate the length of the palindromic sequence, while the Δ*G*, Δ*H*, and Δ*S* were calculated with mFold [42]. The bottom shows the consensus sequence [43, 44] with the labeling positions according to Table 1. Moreover, the center of the dyad symmetry is highlighted in gray while nucleotides in direct contact with PACueR are highlighted in green.

To investigate conformational changes in DNA upon binding of PACueR and Cu(I), we prepared six spin‐labeled DNA sequences derived from the copZ2 and mexPQ‐opmE promoter regions (listed in Table 1). The sequences were chosen based on the affinity between PACueR and the promoter sequences. An earlier publication found five promoter sequences with different affinities correlating with the length of their continuous dyad symmetry. The length of the palindromic sequence is of importance since it correlates with DNA hairpin formation [41]. The longer the palindromic sequence from the center, without mismatches, the higher the resulting free energy upon hairpin formation. Thus, favoring a partially dissociated DNA stretch (Figure 2). Moreover, it is important to note that nucleotides that are in direct contact with PACueR significantly alter the affinity compared to base pairs without direct contact. By EMSA assay, it was revealed that copZ2 with 10 bp dyad symmetry exhibited the highest affinity (33 nM) compared to mexPQ‐opmE with two‐base‐pair palindromic sequence, the second lowest (146 nM) [18]. By selecting these two sequences, we anticipate obtaining a comprehensive view of the mechanism of the transcription initiation of PACueR. The labeling sites were chosen, within the binding site of PACueR, but avoiding base pairs that directly interact with the protein (Figure 2 and Table 1). More specifically, three labeled DNA strands per gene were generated. The first two sequences were labeled at the center and outside of the dyad symmetry while the third sequence carries both labels on the outside (Figure 2 and Table 1). Figures S1–S6 present the mass spectra of all the DNA sequences. Wild‐type (WT) PACueR was expressed and purified using a previously established protocol developed in our lab [40]. Figure S7 shows the SDS‐Gel picture of the purified PACueR. Figure S8 presents the EMSA of all spin‐labeled DNA studied in this research, showing interaction with PACueR.

**TABLE 1:** CopZ2 and mexPQ‐opmE DNA sequences and labeling positions (defined as iAmC2T).

copZ2		
dyad symmetry	5′‐ ggatt **GACCTTGACAccaTGTCAAGGTC** gaaaat‐3′	10 bp
Name code	Sequence and label position	Theoretical distance (nm)^a^
CopZ2_1	5′‐ GGATTGACC/iAmMC2T/TGACACCATGTCAAGGTCGAAAAT‐3′ 5′‐ ATTTTCGACCTTGACA/iAmMC2T/GGTGTCAAGGTCAATCC‐3′	3.2–3.7
CopZ2_2	5′‐ GGATTGACCTTGACACCATGTCAAGGTCGAAAAT‐3′ 5′‐ ATTT/iAmMC2T/CGACCTTGACA/iAmMC2T/GGTGTCAAGGTCAATCC‐3′	4.0–4.5
CopZ2_3	5′‐ GGATTGACC/iAmMC2T/TGACACCATGTCAAGGTCGAAAAT‐3′ 5′‐ ATTTTCGACC/iAmMC2T/TGACATGGTGTCAAGGTCAATCC‐3′	3.9–4.4

** mexPQ‐opmE**		
** dyad symmetry**	5′‐ gggtt **GACCTTG**c**CA**agg**TG**t**CAAGGTC** gataac‐3′	**2 bp**
**Name code**	**Sequence and label position**	** Theoretical distance (nm)** a
MexPQ_1	5′‐ GGGTTGACCTTGCCAAGGTGTCAAGGTCGATAAC‐3′ 5′‐ GTTATCGACC/iAmMC2T/TGACACC/iAmMC2T/TGGCAAGGTCAACCC‐3′	3.2–3.7
MexPQ_2	5′‐ GGGTTGACCTTGCCAAGGTGTCAAGGTCGATAAC‐3′ 5′‐ GTTA/iAmMC2T/CGACCTTGACACC/iAmMC2T/TGGCAAGGTCAACCC‐3′	4.3–4.8
MexPQ_3	5′‐ GGGTTGACC/iAmMC2T/TGCCAAGGTGTCAAGGTCGATAAC‐3′ 5′‐ GTTATCGACC/iAmMC2T/TGACACCTTGGCAAGGTCAACCC‐3′	4.0–4.5

a Theoretical distance was obtained by measuring the distance between the two nitrogen atoms of the spin labels of the modeled promoter sequence containing the spin labels. The underlined sequence reflects the PACueR binding site, and the bold letters show the palindromic sequence, the bp were calculated by the length of the continuous palindromic sequence from the center outward.

**FIGURE 3:**
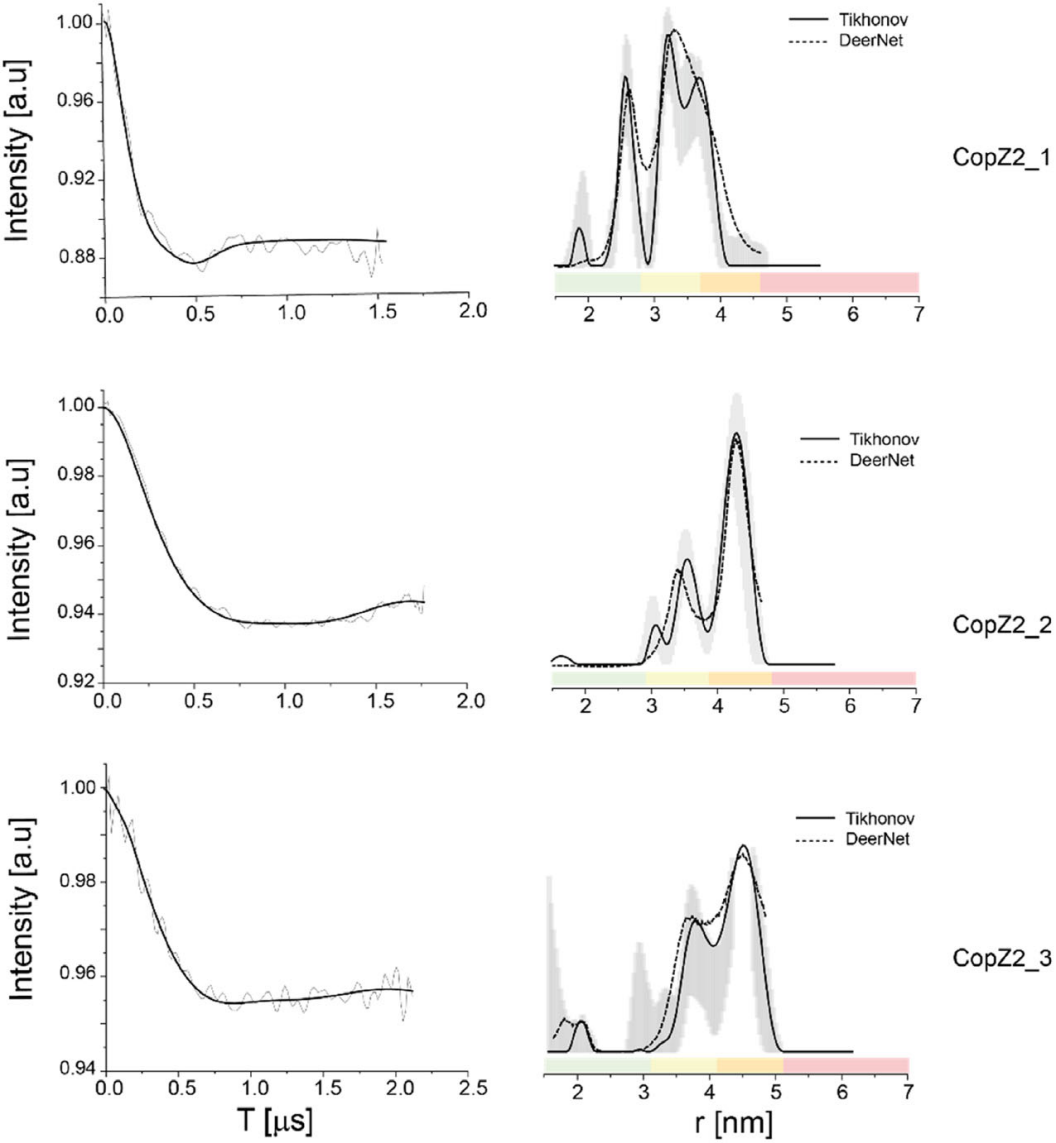
DEER on spin‐labeled copZ2 DNA: Q‐band DEER time domain signals after background subtraction and the corresponding distance distribution function for three copZ2 DNA sequences. The data was analyzed using the DeerAnalysis program using Tikhonov regularization, where the regularization parameter was 20 (solid black lines) and using DEERNet (dashed black lines). Distance distribution validation considered white noise, background start and dimensionality. The color bar indicates reliability ranges (green: shape reliable; yellow: mean and width reliable; orange: mean reliable; pink: no quantification possible).

**FIGURE 4:**
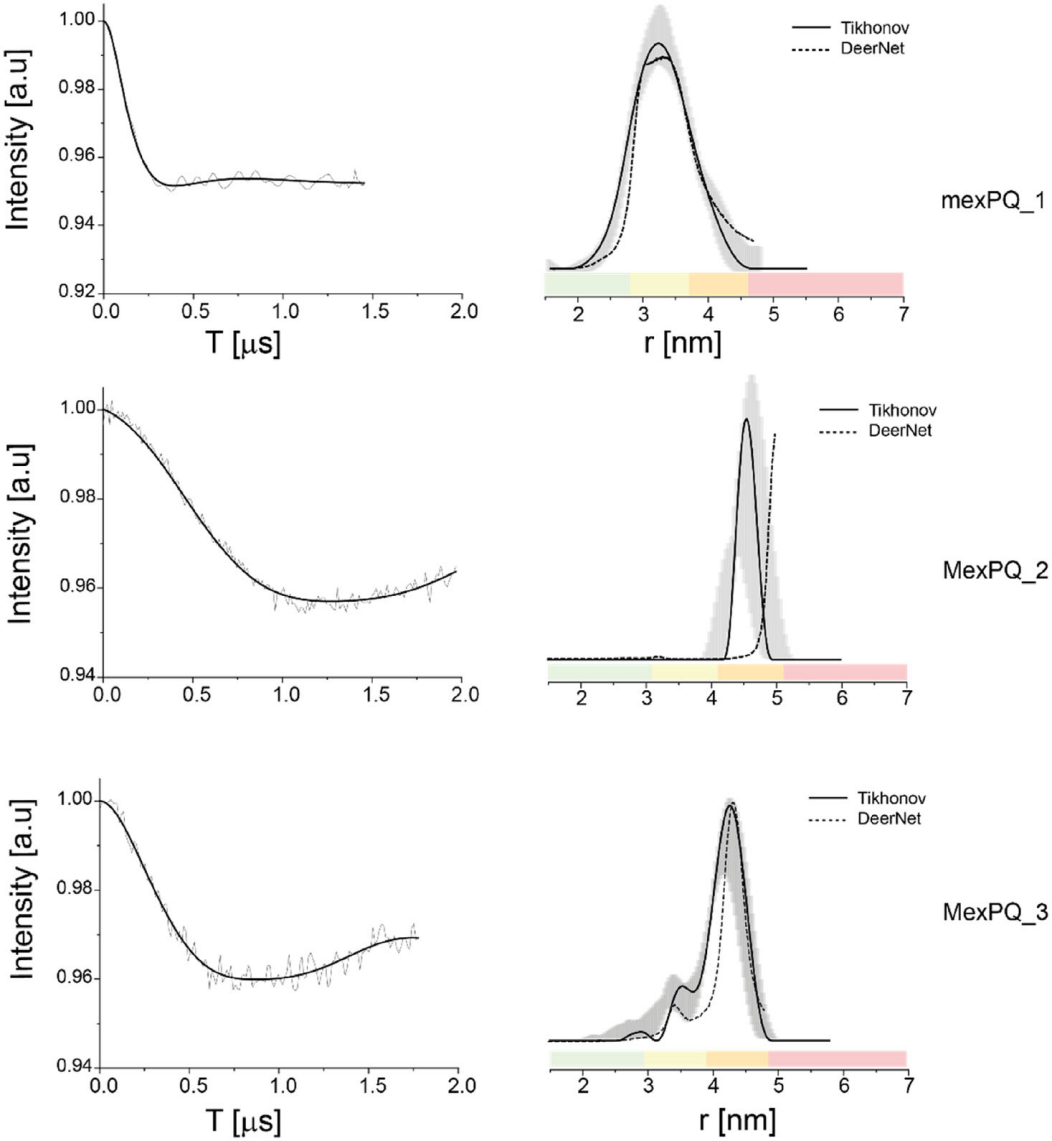
DEER on spin‐labeled mexPQ‐opmE DNA: Q‐band DEER time domain signals after background subtraction and the corresponding distance distribution function for three mexPQ‐opmE DNA sequences. The data was analyzed using the DeerAnalysis program using Tikhonov regularization, where the regularization parameter was 20 (solid black lines) and using DEERNet (dashed black lines). Distance distribution validation considered white noise, background start, and dimensionality. The color bar indicates reliability ranges (green: shape reliable; yellow: mean and width reliable; orange: mean reliable; pink: no quantification possible).

Pulsed electron–electron double resonance (DEER) measurements were performed at Q‐band frequency (34 GHz) to determine distance distributions between paramagnetic centers. DEER spectroscopy, widely used in biophysical research with nitroxide spin labels, enables not only the determination of average distances between spin labels but also provides insight into the width of the distance distribution, reflecting molecular flexibility and conformational heterogeneity [45]. Figure 3 displays the DEER signals along with the corresponding distance distribution functions for three copZ2 DNA sequences. Similarly, Figure 4 presents the DEER data for three mexPQ‐opmE sequences. The experimentally observed average spin‐to‐spin distances are in good agreement with the theoretical distances derived from the modeled structures of the DNA promoters containing spin labels, as summarized in Table 1. This agreement underscores the consistency between structural predictions and DEER‐based experimental data.

**FIGURE 5:**
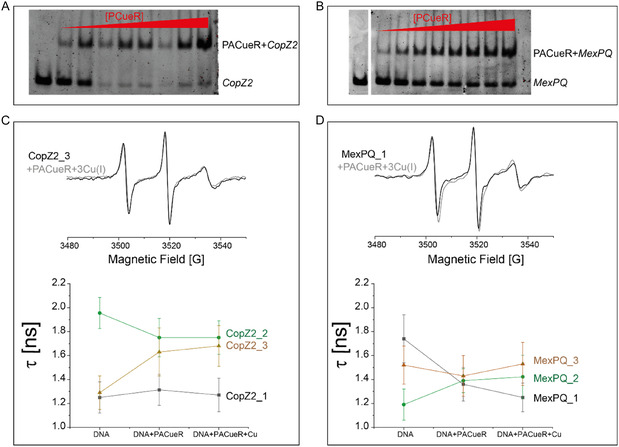
EMSA gel pictures: complex formation as a function of PACueR concentration for (A) copZ2 promotor, and (B) mexPQ‐opmE promotor. Room‐temperature CW‐EPR data for the three spin‐labeled DNA sequences of (C) CopZ2 DNA, and (D) MexPQ‐OpmE DNA. The tumbling rate (*τ_c_*) for each spectrum was calculated using equation (1).

We then explored the changes in the dynamics and structure of the DNA upon Cu(I) and PACueR binding for each of the spin‐labeled DNA. Figure 5 shows the EMSA of both DNA sequences as a function of PACueR concentration. The data suggests that PACueR has higher affinity to copZ2 DNA than to mexPQ‐opmE DNA; PACueR binds to both DNA sequences. We first ran room temperature (RT) CW‐EPR experiments (Figure 5). The CW‐EPR spectra of the spin‐labeled DNA is characterized by high dynamics of the spin‐label. The correlation time (*τ*_c_) of the spin‐labeled DNA was estimated by using the following equation [46]:

(1) $$\tau_{c}=\left(6.51\times 10^{-10}\right)\text{Δ}H_{0}\left\{{\left[\frac{h\left(0\right)}{h\left(-1\right)}\right]}^{\frac{1}{2}}+{\left[\frac{h\left(0\right)}{h\left(1\right)}\right]}^{\frac{1}{2}}-2\right\}\sec$$

where Δ*H*_0_ is the peak‐to‐peak line width of the *M*_I _= 0 component (the central peak) in Gauss, and *h*(−1), *h*(0), and *h*(1) are the peak‐to‐peak heights of the *M*_I _= 1,0,–1 (*M*_I_ refers to the nuclei ^14^N spin state) lines, respectively. The correlation time was determined to be in the range of 1.0–2.0 ns, indicating rapid molecular motion. Only minor alterations in the dynamic behavior were observed upon binding of Cu(I) and DNA, suggesting that the spin‐label remains highly flexible, even when the DNA is complexed with the protein. This implies that the local environment of the spin‐label is not significantly restricted by either metal coordination or protein–DNA interactions.

**FIGURE 6:**
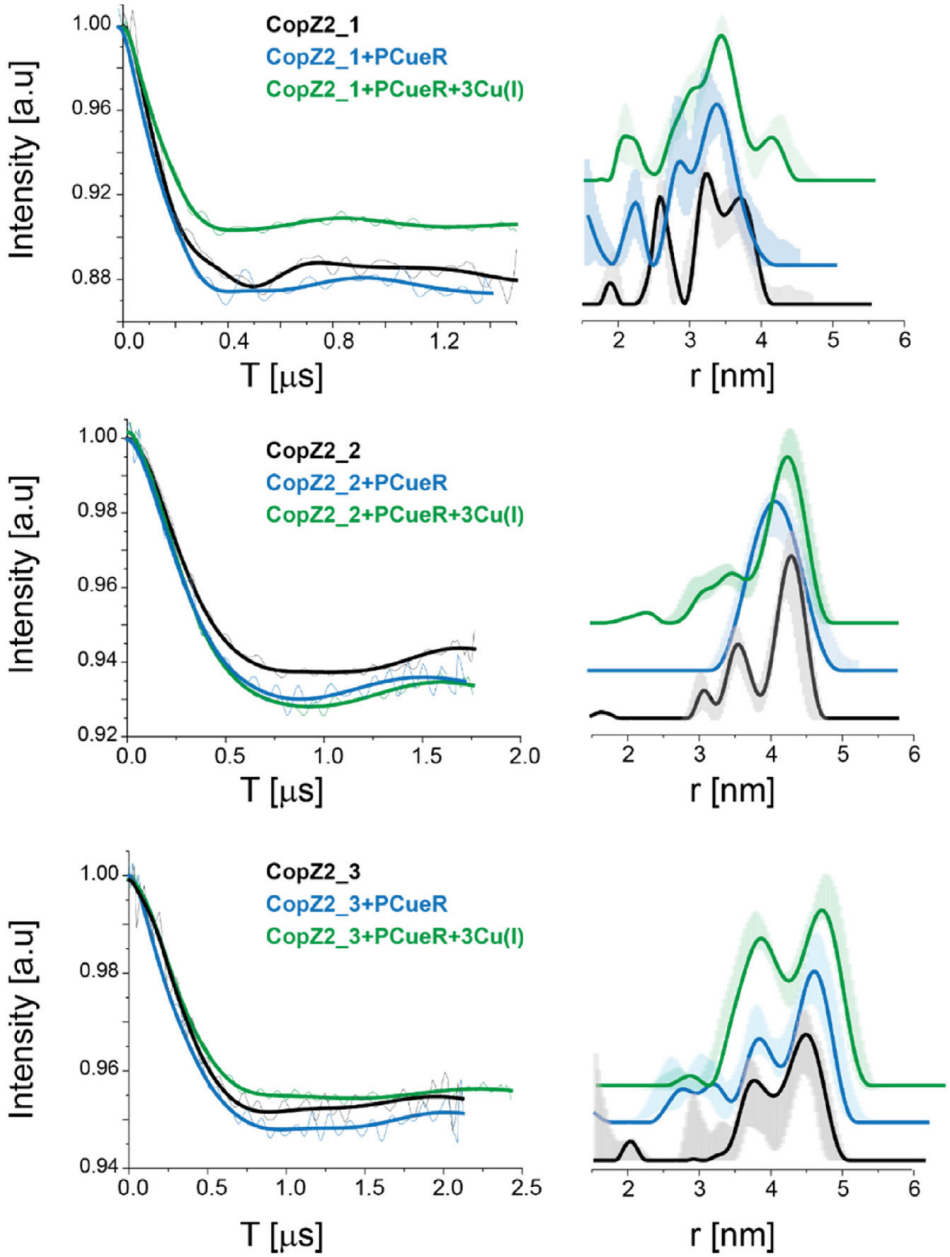
DEER on spin‐labeled copZ2 DNA as a function of PACueR and Cu(I) binding: Q‐band DEER time domain signals after background subtraction and the corresponding distance distribution function for three copZ2 DNA (black line), in the presence of PACueR (blue line), and PACueR and Cu(I) (green line). The data was analyzed using the DeerAnalysis program using Tikhonov regularization, where the regularization parameter was 20. Distance distribution validation considered white noise, background start and dimensionality.

**FIGURE 7:**
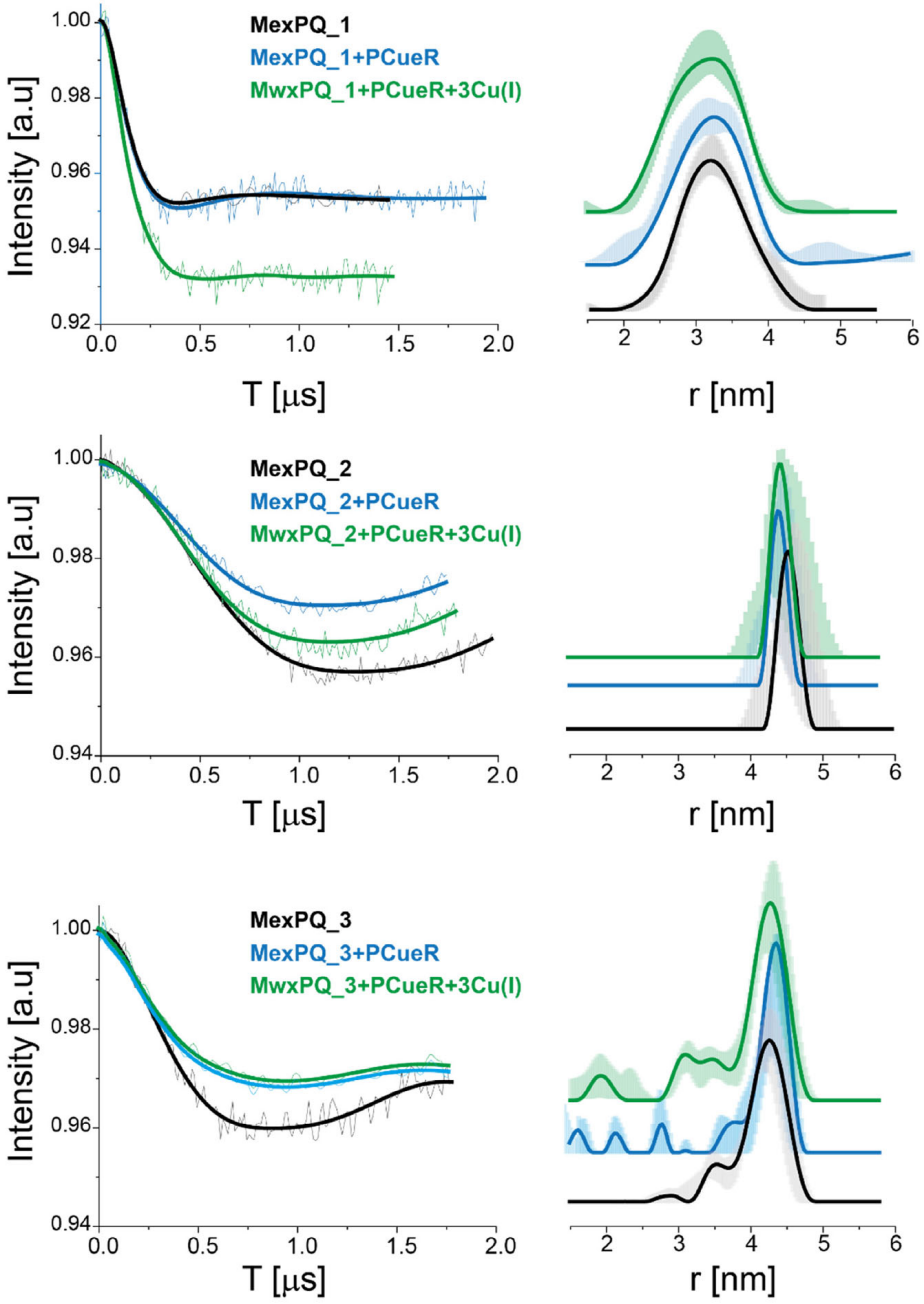
DEER on spin‐labeled mexPQ‐opmE DNA as a function of PACueR and Cu(I) binding: Q‐band DEER time domain signals after background subtraction and the corresponding distance distribution function for three mexPQ‐opmE DNA (black line), in the presence of PACueR (blue line), and PACueR and Cu(I) (green line). The data was analyzed using the DeerAnalysis program using Tikhonov regularization, where the regularization parameter was 20. Distance distribution validation considered white noise, background start and dimensionality.

Next, DEER measurements were performed on each DNA sequence as a function of Cu(I) and PACueR binding (Figures 6 and 7). DEER raw data without background subtraction is presented in Figures S9–S14. The DEER data on copZ2 promoters indicates conformational changes in the DNA as a function of Cu(I) and DNA binding. However, only minor conformational changes were detected for mexPQ‐opmE DNA. This suggests that even if PACueR can bind to mexPQ‐opmE DNA, it cannot necessarily report on transcription termination or activation, it thus hints toward a fine tune mechanism of transcription based on the sensitivity of the protein to the promoter.

A more detailed examination of the conformational changes occurring at the copZ2 promoter reveals specific structural rearrangements upon PACueR and Cu(I) binding. For the CopZ2_1 construct, in the unbound state, there are two distributions, one very narrow around 2.6 ± 0.1 nm and other around 3.5 ± 0.5 nm. Upon binding of PACueR, the short‐distance distribution disappears, and a broad distance distribution appears around 3.3 ± 0.5 nm, indicating a compaction or bending of the DNA at this site. Interestingly, when Cu(I) is subsequently introduced, this distance increases to 3.6 ± 0.5 nm, suggesting a partial relaxation of the DNA or some separation between the two DNA strands induced by copper binding. A similar trend is observed for CopZ2_2, where the mean distance decreases from 4.4 ± 0.3 nm to 4.1 ± 0.5 nm following PACueR binding, again indicative of DNA bending or structural tightening. Upon addition of Cu(I), however, the distance returns to approximately its original value (4.4  ±  0.3 nm), suggesting that copper binding counteracts or modifies the conformational change induced by PACueR alone. CopZ2_3 constructs display a bimodal distance distribution, one around 3.7 nm and the second around 4.5 nm in the unbound state. Upon PACueR and Cu(I) binding, there is a shift of the second distribution to 4.7 nm (upon PACueR binding) and 4.8 nm (upon PACueR and Cu(I) binding). Notably, the proportion of the shorter‐distance population (3.7 nm) increases upon addition of Cu(I). Both CopZ2_1 and CopZ2_3 constructs are characterized by spin‐labels at two different DNA strands, therefore they are characterized by more distributions as compared to CopZ2_2. The change in the various distribution as a function of Cu(I) and PACueR indicates that there is not only a change in the whole DNA bending, but also a change in the distance between the two DNA strands.

The comparison between the two promoter sequences reveals that copZ2 exhibits more pronounced changes in signal than mexPQ‐opmE under both PACueR binding and Cu(I) addition (Figures 6 and 7). The smaller change in the EPR signature for mexPQ‐opmE can be attributed to its lower affinity for PACueR. Consistent with this, promoter sequences with higher PACueR affinity display up to 50‐fold greater expression levels than mexPQ‐opmE upon copper exposure. These trends match the overall signal differences observed between the CopZ2_3 mutant and MexPQ_3. Both sequences were spin‐labeled outside the dyad symmetry center. In both sequences, copper exposure leads to an increased population of the shorter distance distribution of ≈3.7 nm for CopZ2_3 and 3.2 nm for MexPQ_3 representing a reduction of about 1 nm compared to the longer distance distribution in the absence of copper. In line with earlier observations, the population of this short‐distance distribution in the active MexPQ_3 sequence (with PACueR and Cu(I)) is markedly lower than in active CopZ2_3, reflecting the reduced formation of DNA–protein complexes due to the lower binding affinity.

Overall, the EPR findings demonstrate that PACueR binding induces localized bending and structural changes in the copZ2 promoter region, which are further modulated upon Cu(I) binding.

## 3 Discussion

Transcription factors are central to nearly all cellular functions, ranging from development and differentiation to environmental sensing and preserving cellular identity. Transcription mechanisms are linked to a vast array of pathologies, including cancers, autoimmune diseases, and neurological disorders. In this study, we focused on the *P. aeruginosa* metalloregulator protein CueR, a copper‐responsive transcription factor. Upon exposure to elevated Cu(I) levels, PACueR activates the transcription of at least five genes: copZ1, copZ2, mexPQ‐opmE, copA1, and PA3515 to PA3519. All known proteins are key players in copper homeostasis or otherwise detoxification. Our aim was to unravel the biophysical and structural responses of CueR when it interacts with two specific promoters: copZ2 (encoding a copper chaperone) and MexPQ‐opmE (encoding part of an RND‐family efflux pump) [3, 47, 48]. We employed site‐directed spin labeling on the promoters at multiple positions, followed by pulsed DEER experiments as a function of PACueR and Cu(I). For the copZ2 promoter, we observed clear conformational changes in the DNA upon PACueR and copper binding. In contrast, the mexPQ‐opmE promoter showed only subtle but identical structural shifts under the same conditions. These observations are consistent with previous EPR findings on the PACueR protein which showed that PACueR undergoes more pronounced changes in dynamics when bound to the copZ2 promoter versus mexPQ‐opmE [40]. Consistently, EMSA results demonstrated a higher binding affinity of PACueR for the copZ2 promoter over mexPQ‐opmE. Further transcriptomic analyses supports this mechanistic framework: under copper stress, copZ2 promoter activity and correspondingly mRNA expression dramatically increase (by tens of thousands of reads), whereas induction of mexPQ‐opmE is comparatively lower [38]. From a physiological standpoint, this differential regulation is functionally intuitive: CopZ2, a metallochaperone, is rapidly upregulated to buffer cytoplasmic copper during stress, while MexPQ‐opmE, an efflux system targeting potentially toxic organic compounds, is activated to a lesser extent [3]. Moreover, our findings indicate that transcription initiation in PACueR regulated genes is governed primarily by the affinity and length of the palindromic promoter sequence rather than by CueR itself. While the underlying initiation mechanism appears conserved for CueR and across these genes, differences in promoter affinity serve as fine tuning mechanism, modulating transcription levels in response to copper.

EPR spectroscopy, particularly through site‐directed spin labeling and pulsed DEER techniques, proves to be once again a powerful biophysical tool for dissecting the structural and mechanistic intricacies of transcription factor‐DNA interactions [29]. In the case of the copper‐responsive transcription factor CueR from *P. aeruginosa*, EPR enabled the detection of subtle and promoter sequence‐specific conformational changes upon protein and metal ion binding. The structural alterations observed in the copZ2 promoter, contrasting the subtle changes seen in mexPQ‐opmE, highlight how EPR can resolve differential DNA dynamics that correlate with functional outcomes, such as promoter affinity and transcriptional activation. These insights, unattainable through conventional biochemical assays alone, underscore EPR's unique ability to capture real‐time, nanoscale changes in biomolecular complexes, thereby offering a mechanistic view of metalloproteins that regulate transcription in response to cellular signals.

## 4 Conclusion

Herein, we integrated EPR experiments together with biochemical data to show that PACueR potentially enhances a more substantial expression response for copZ2 than for mexPQ‐opmE under copper stress, which aligns with the functional roles of these proteins in metal detoxification as well as with transcription data obtained from other studies. This work highlights the importance of EPR as a biophysical tool to understand complicated biological mechanisms. By employing EPR measurements, we confirmed earlier findings that the underlying initiation mechanism is conserved for CueR across these genes. However, variations in promoter affinity and the length of the dyad symmetry act as fine‐tuning factors that modulate transcription levels in response to copper. In this context, it is not the regulatory protein itself that dictates expression levels, but rather the metal ion and DNA promoter sequence itself.

## 5 Experimental Methods

### 5.1 Expression and Purification of *P. aeruginosa* CueR (PACueR)

Wild‐type (WT) PACueR construct was amplified by PCR and inserted into a modified pET28a expression vector. The integrity and correctness of the recombinant plasmids were verified through DNA sequencing. To facilitate purification, a maltose‐binding protein (MBP, expressed in the lab) tag, along with a TEV (expressed in the lab) protease cleavage site, was engineered upstream of the protein's start codon. Following transformation into *E.* *coli* BL21 cells, protein expression was carried out in a total of 2 L of LB medium, distributed across five 1‐L flasks, each containing 400 mL of culture. The cells were incubated at 37°C until the optical density at 600 nm (OD_600_) reached at least 0.8, after which 200 μL of 1 mM IPTG was added to each flask to induce expression. Cultures were then grown overnight at 22°C. Cells were harvested by centrifugation at 8000 rpm for 25 min at 4°C, and the resulting pellets were resuspended in lysis buffer composed of 25 mM Tris–HCl (pH 8.5), 250 mM NaCl, 1% Triton X‐100, and 20 mM imidazole. The suspension was supplemented with 1 mM dithiothreitol (DTT) and was homogenized. Cell disruption was achieved via sonication using 10‐s pulses with 10 sec intervals, at 35% amplitude for a total of 10 min. Lysates were clarified by centrifugation at 14,000 rpm for 25 min at 4°C. The supernatant was then incubated with 5 mL of Ni‐NTA resin to capture the His‐tagged fusion protein. After extensive washing and elution, protein presence and purity were assessed by SDS‐PAGE and Coomassie Blue staining. To remove the MBP tag, the eluted protein was digested with TEV protease (2 mg/mL); digestion was performed overnight at 17°C in a 1 L dialysis buffer (25 mM Tris–HCl pH 8.5, 250 mM NaCl, 0.1% Triton X‐100) with gentle agitation using a dialysis bag.

### 5.2 DNA Spin‐Labeling Protocol

Modified DNA containing a thymine base substitution was obtained from IDT (via Syntezza Bioscience Ltd, Israel) as HPLC‐purified oligonucleotides and dissolved in double‐distilled water (DDW) to prepare a 100 µM stock solution. Ethanol precipitation was performed by freezing in liquid nitrogen using an Eppendorf tube. Specifically, 20 µL of the DNA solution (2 nmol) was combined with 2 µL of 3 M sodium acetate (NaOAc), pH 5.2, followed by the addition of 60 µL of ice‐cold ethanol (stored at –20°C). The mixture was incubated while floating in liquid nitrogen for 1 h. Afterward, the sample was centrifuged at 14,000 rpm for 25 min at 4°C, yielding a visible white pellet indicative of successful DNA precipitation. The supernatant was carefully removed without disturbing the pellet, which was then dried overnight using either lyophilization or a SpeedVac.

For the labeling step, 10 µL of labeling buffer (0.1 M sodium tetraborate, pH 8.5) was added to the pellet. To this, 8 µL of a 4 mM solution of nitroxide‐NHS‐ester (prepared in dry DMSO) was introduced and gently mixed by pipetting. The labeling reaction was carried out at 50°C for 1 h with shaking at 500 rpm. To quench the reaction, 1.5 µL of 3 M NaCl and 50 µL of cold ethanol were added, and the DNA was reprecipitated using the same liquid nitrogen protocol as before. After centrifugation under identical conditions (14,000 rpm, 25 min, 4°C), the supernatant was removed, and the pellet was again dried overnight. The dried pellet was dissolved in 50 µL of triethylammonium acetate (TEAA) buffer and purified using reverse‐phase HPLC with an XBridge BEH C18 column (130 Å, 2.5 µm, 4.6 × 50 mm; Waters) on an Agilent 1200 system. The 50 µL sample was loaded and separated with the following gradient profile: 100% buffer A (0.1 M TEAA, pH 6.5 with 5% acetonitrile) for 10 min (isocratic), followed by a linear gradient from 100% buffer A to 85% buffer A and 15% buffer B (100% acetonitrile) from 10 to 15 min. The gradient continued from 85% to 0% buffer A (100% buffer B) from 15 to 20 min, held at 100% buffer B for 2 min, then transitioned back to 100% buffer A over 3 min, and held at 100% buffer A for an additional 2 min. Flow rate was maintained at 1 mL/min.

Fractions containing the labeled DNA were collected and dried via lyophilization or SpeedVac. The labeled oligonucleotides were then reconstituted in water and mixed with an equimolar amount of complementary strand. Annealing was performed by thermocycling the mixture three times from 90°C to 12°C over 18 min to ensure duplex formation. The resulting double‐stranded DNA was analyzed by LC‐MS/MS to verify successful spin labeling. Finally, the labeled duplex DNA was stored at –20°C, and all EPR measurements were conducted using DNA concentrations ranging from 20 to 50 µM.

### 5.3 Cu(I) Addition for EPR Measurements

Cu(I) (Tetrakis (acetonitrile)copper(I) hexafluorophosphate) was added to the protein solution under nitrogen gas to preserve anaerobic conditions. No Cu(II) EPR signal was observed at any time.

### 5.4 EPR Measurements

CW‐EPR spectra were recorded using an E500 Elexsys Bruker spectrometer operating at 9.4–9.5 GHz equipped with Elexsys super high sensitivity probehead. The spectra were recorded at RT at microwave power of 20.0 mw, modulation amplitude of 1.0 G, time constant of 60 ms, and a receiver gain of 60 dB. The samples were measured in 0.8 mm capillary quartz tubes (vitrocom).

### 5.5 Double Electron Electron Resonance (DEER) Measurements

The DEER experiment, *π*/2(*ν*_obs_) − *τ*_1_ − *π*(*ν*_obs_) − *t*′ − *π*(*ν*_pump_) − (*τ*_1_ + *τ*_2_ − *t*′) − *π*(*ν*_obs_) − *τ*_2_ − echo, was carried out at 50 ± 0.5 K on a Q‐band Elexsys E580 spectrometer (equipped with a 2‐mm probe head) and 50 W AmpQ. A two‐step phase cycle was employed on the first pulse. The echo was measured as a function of *t*′, whereas *τ*_2_ was kept constant to eliminate relaxation effects. The durations of the observer *π*/2 and *π* pulses were 12 ns and 24 ns, respectively. The duration of the *π* pump pulse was 24 ns, and the dwell time was 16 ns. *τ*_1_ was set to 200 ns. The observer frequency was 34.05 GHz pump frequency: 34.12 GHz magnetic field: 12040 G. The samples were measured in 1.6‐mm capillary quartz tubes (Wilmad‐LabGlass). The data was analyzed using the DeerAnalysis 2019 program [49]. Both Tikhonov regularization and DeerNet were used to analyze the data [50]. Tikhonov validation considers White noise, background start, and background dimensionality. 20% glycerol was added to all samples.

### 5.6 Electrophoretic Mobility Shift Assay (EMSA) With EtBr Stain

EMSA experiments were carried out to detect PACueR interactions with the various DNAs at RT for about 30 min in suitable binding buffer (25 mM Tris‐HCl pH = 8.5, 250 mM NaCl, 5% Glycerol). The gel was run with TAE buffer (40 mM Tris‐HCl, 20 mM acetic acid, 1 mM EDTA) at 4°C and 80V for 1 h. The gel was stained using 1 µg/mL ethidium bromide for 15 min and analyzed with a Gel Doc EZ BioRad system.

## Supporting Information

Additional supporting information can be found online in the Supporting Information section. **Supporting Fig. S1:** Mass spectrometry data of non‐labeled DNA CopZ2_1 (1^st^ strand = 10,572 Da; 2^nd^ strand = 10,505 Da) and labeled DNA CopZ2_1 (1^st^ strand = 10,739 Da; 2^nd^ strand = 10671 Da). **Supporting Fig. S2:** Mass spectrometry data of non‐labeled DNA CopZ2_1 (2^nd^ strand = 10,463 Da) and labeled DNA CopZ2_2 (2^nd^ strand = 10,925 Da). **Supporting Fig. S3:** Mass spectrometry data of non‐labeled and labeled DNA CopZ2_3. **Supporting Fig. S4:** Mass spectrometry data of non‐labeled and labeled DNA MexPQ_1. **Supporting Fig. S5:** Mass spectrometry data of non‐labeled and labeled DNA MexPQ_2. **Supporting Fig. S6:** Mass spectrometry data of non‐labeled and labeled DNA MexPQ_3. **Supporting Fig. S7:** SDS‐Gel Picture of purified WT_PACueR. **Supporting Fig. S8:** EMSA pictures: complex formation of all spin‐labeled DNA promoters used in this study as a function of PACueR and Cu(I) binding. **Supporting Fig. S9:** DEER on spin‐labeled CopZ2_1 DNA as a function of PACueR and Cu(I) binding: Q‐band DEER time domain signals before (left side) and after background subtraction (intermediate) and the corresponding distance distribution function for CopZ2_1 DNA, in the presence of PACueR, and PACueR and Cu(I). The data was analyzed using the DeerAnalysis program using Tikhonov regularization, where the regularization parameter was 20 (solid black lines) and using DEERNet (dashed black lines). Distance distribution validation considered white noise, background start and dimensionality. The color bar indicates reliability ranges (green: shape reliable; yellow: mean and width reliable; orange: mean reliable; pink: no quantification possible). **Supporting Fig. S10:** DEER on spin‐labeled CopZ2_2 DNA as a function of PACueR and Cu(I) binding: Q‐band DEER time domain signals before (left side) and after background subtraction (intermediate) and the corresponding distance distribution function for CopZ2_2 DNA, in the presence of PACueR, and PACueR and Cu(I). The data was analyzed using the DeerAnalysis program using Tikhonov regularization, where the regularization parameter was 20 (solid black lines) and using DEERNet (dashed black lines). Distance distribution validation considered white noise, background start and dimensionality. The color bar indicates reliability ranges (green: shape reliable; yellow: mean and width reliable; orange: mean reliable; pink: no quantification possible). **Supporting Fig. S11:** DEER on spin‐labeled CopZ2_3 DNA as a function of PACueR and Cu(I) binding: Q‐band DEER time domain signals before (left side) and after background subtraction (intermediate) and the corresponding distance distribution function for CopZ2_3 DNA, in the presence of PACueR, and PACueR and Cu(I). The data was analyzed using the DeerAnalysis program using Tikhonov regularization, where the regularization parameter was 20 (solid black lines) and using DEERNet (dashed black lines). Distance distribution validation considered white noise, background start and dimensionality. The color bar indicates reliability ranges (green: shape reliable; yellow: mean and width reliable; orange: mean reliable; pink: no quantification possible). **Supporting Fig. S12:** DEER on spin‐labeled MexPQ_1 DNA as a function of PACueR and Cu(I) binding: Q‐band DEER time domain signals before (left side) and after background subtraction (intermediate) and the corresponding distance distribution function for MexPQ_1 DNA, in the presence of PACueR, and PACueR and Cu(I). The data was analyzed using the DeerAnalysis program using Tikhonov regularization, where the regularization parameter was 20 (solid black lines) and using DEERNet (dashed black lines). Distance distribution validation considered white noise, background start and dimensionality. The color bar indicates reliability ranges (green: shape reliable; yellow: mean and width reliable; orange: mean reliable; pink: no quantification possible). **Supporting Fig. S13:** DEER on spin‐labeled MexPQ_2 DNA as a function of PACueR and Cu(I) binding: Q‐band DEER time domain signals before (left side) and after background subtraction (intermediate) and the corresponding distance distribution function for MexPQ_2 DNA, in the presence of PACueR, and PACueR and Cu(I). The data was analyzed using the DeerAnalysis program using Tikhonov regularization, where the regularization parameter was 20 (solid black lines) and using DEERNet (dashed black lines). Distance distribution validation considered white noise, background start and dimensionality. The color bar indicates reliability ranges (green: shape reliable; yellow: mean and width reliable; orange: mean reliable; pink: no quantification possible). **Supporting Fig. S14:** DEER on spin‐labeled MexPQ_3 DNA as a function of PACueR and Cu(I) binding: Q‐band DEER time domain signals before (left side) and after background subtraction (intermediate) and the corresponding distance distribution function for MexPQ_3 DNA, in the presence of PACueR, and PACueR and Cu(I). The data was analyzed using the DeerAnalysis program using Tikhonov regularization, where the regularization parameter was 20 (solid black lines) and using DEERNet (dashed black lines). Distance distribution validation considered white noise, background start and dimensionality. The color bar indicates reliability ranges (green: shape reliable; yellow: mean and width reliable; orange: mean reliable; pink: no quantification possible).

## Funding

The study was supported by the BSF‐NSF 2019723 grant.

## Conflicts of Interest

The authors declare no conflicts of interest.

## Supporting information

Supplementary Material
