# Supplementary material for: Electron Paramagnetic Resonance Spectroscopy Reveals Promoter Dependent Transcription Regulation by Copper Activated CueR in Pseudomonas aeruginosa
Source: Chemphyschem. 2026 Jan 25;27(2):e202500625. doi: 10.1002/cphc.202500625 (PMC12833586; doi:10.1002/cphc.202500625)
Supplement: Supplementary file 1 — Supplementary Material [file CPHC-27-e202500625-s001.pdf]

EPR Spectroscopy Reveals Promoter Dependent Transcription Regulation by Copper Activated CueR in *Pseudomonas aeruginosa*

Ameer Yasin[a,+], Misan Irshed[a,+], Lukas Hoffman[a], Yulia Shenberger[a], Lada Gevorkyan-Airapetov[a], Sharon Ruthstein[a,*]

[a] The Chemistry Department and the Institute of Advanced Materials and Nanotechnology, Faculty of Exact Sciences, Bar-Ilan University, Ramat-Gan, Isreal 5290002.

[+] Equal Contribution

[*] sharon.ruthstein@biu.ac.il, +972-3-7384329

SUPPORTING INFORMATION

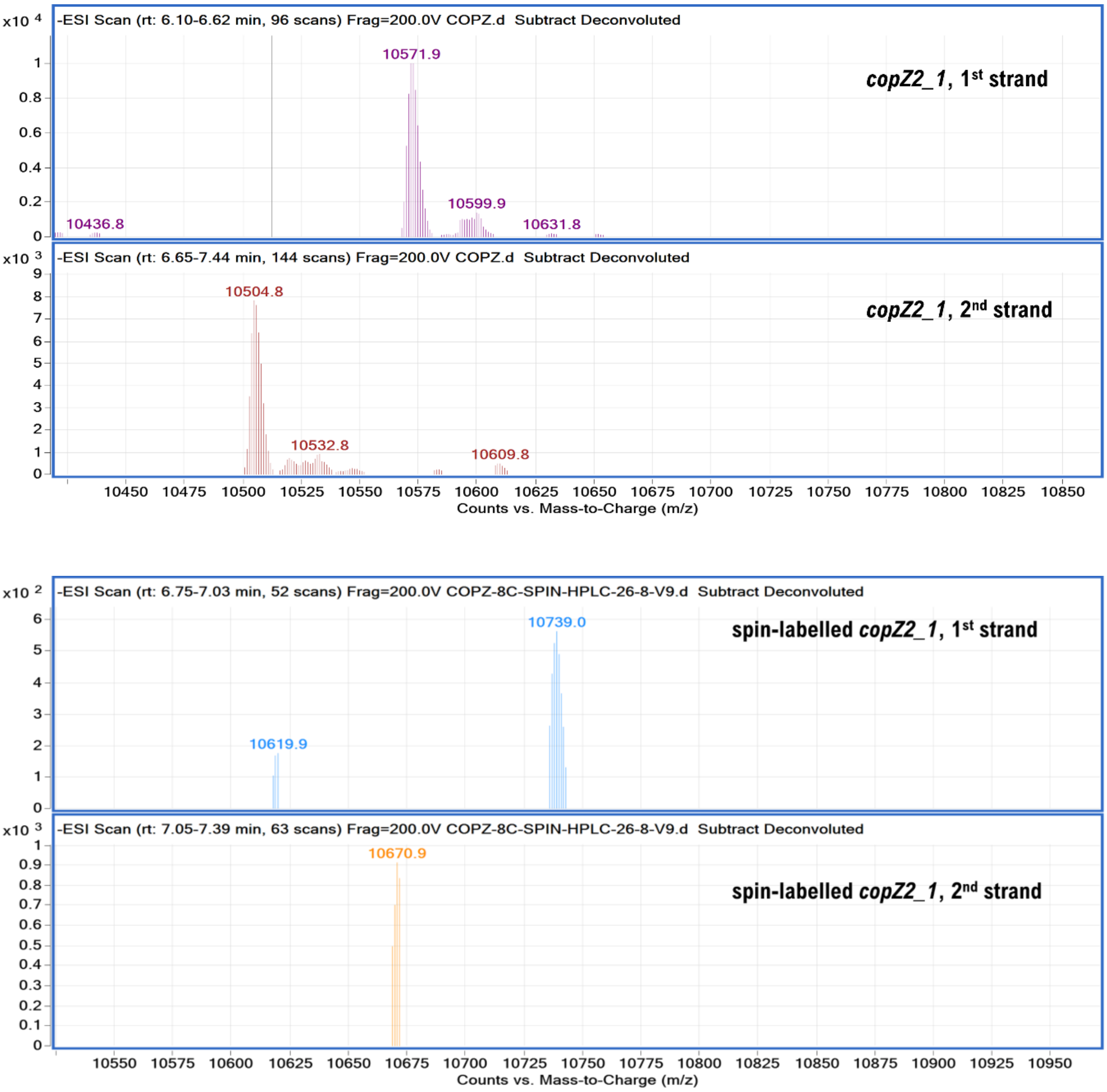

Figure S1: Mass spectrometry data of non-labelled DNA CopZ2_1 (1st strand = 10572 Da; 2nd strand = 10505 Da) and labelled DNA CopZ2_1 (1st strand = 10739 Da; 2nd strand = 10671 Da).

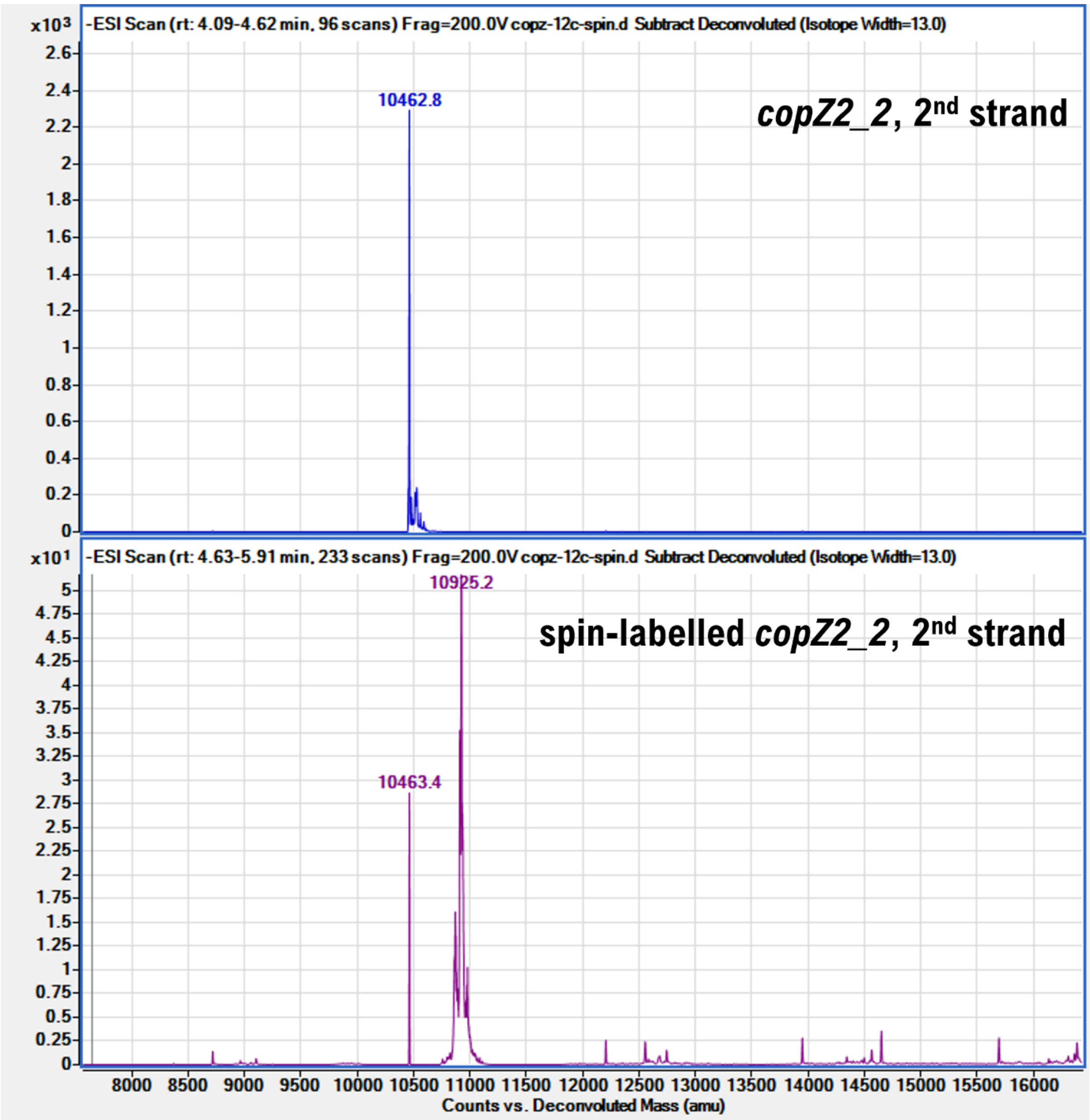

Figure S2: Mass spectrometry data of non-labelled DNA CopZ2_1 (2nd strand = 10463 Da) and labelled DNA CopZ2_2 (2nd strand = 10925 Da).

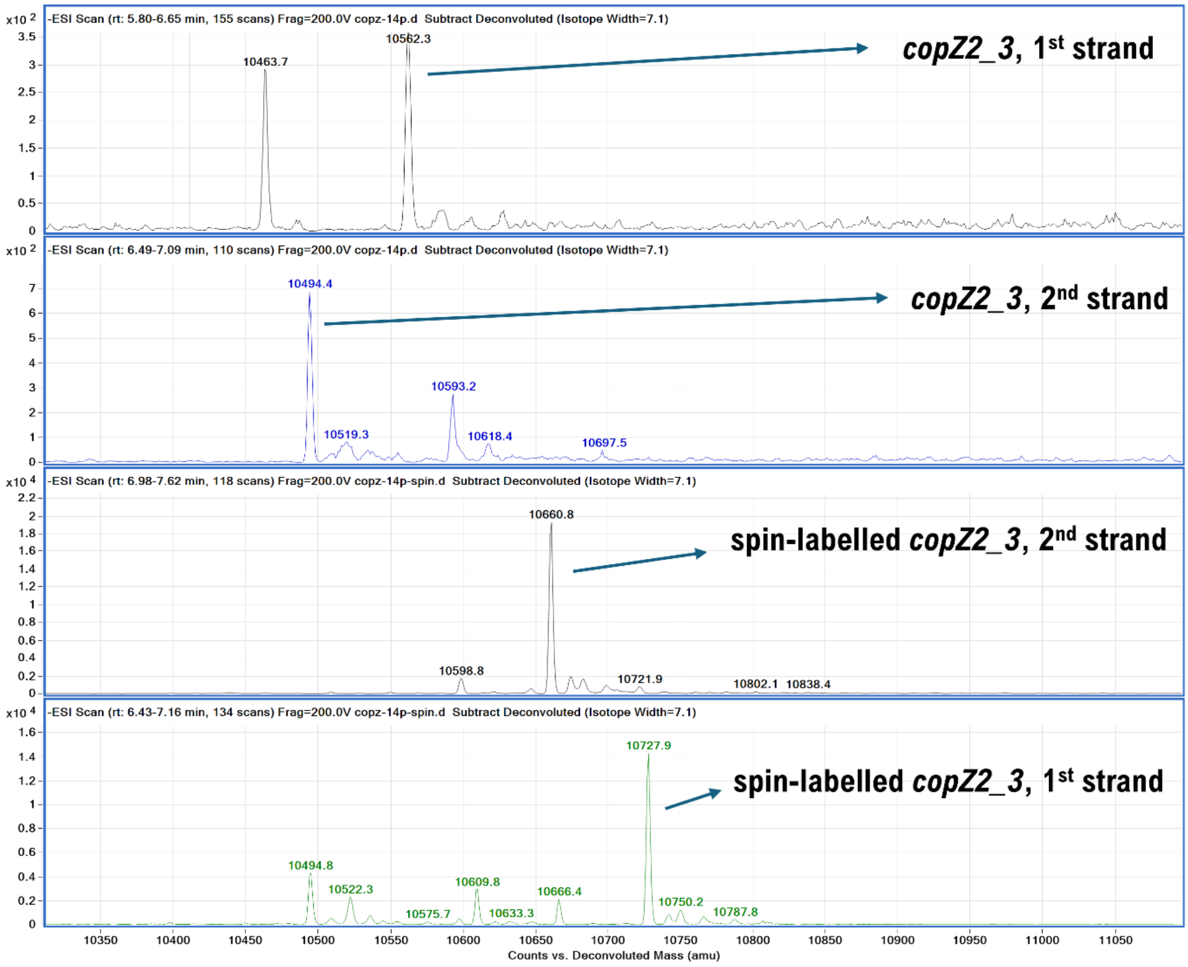

Figure S3: Mass spectrometry data of non-labelled and labelled DNA CopZ2_3.

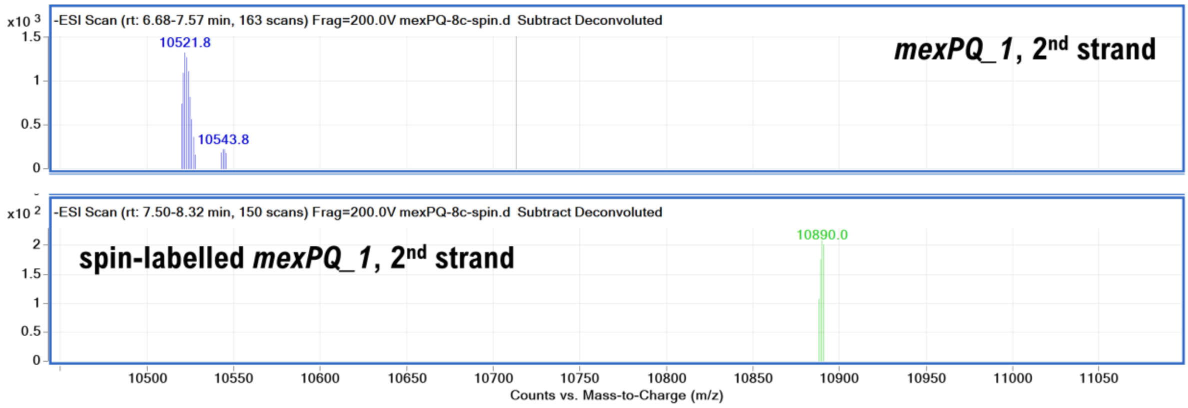

Figure S4: Mass spectrometry data of non-labelled and labelled DNA MexPQ_1.

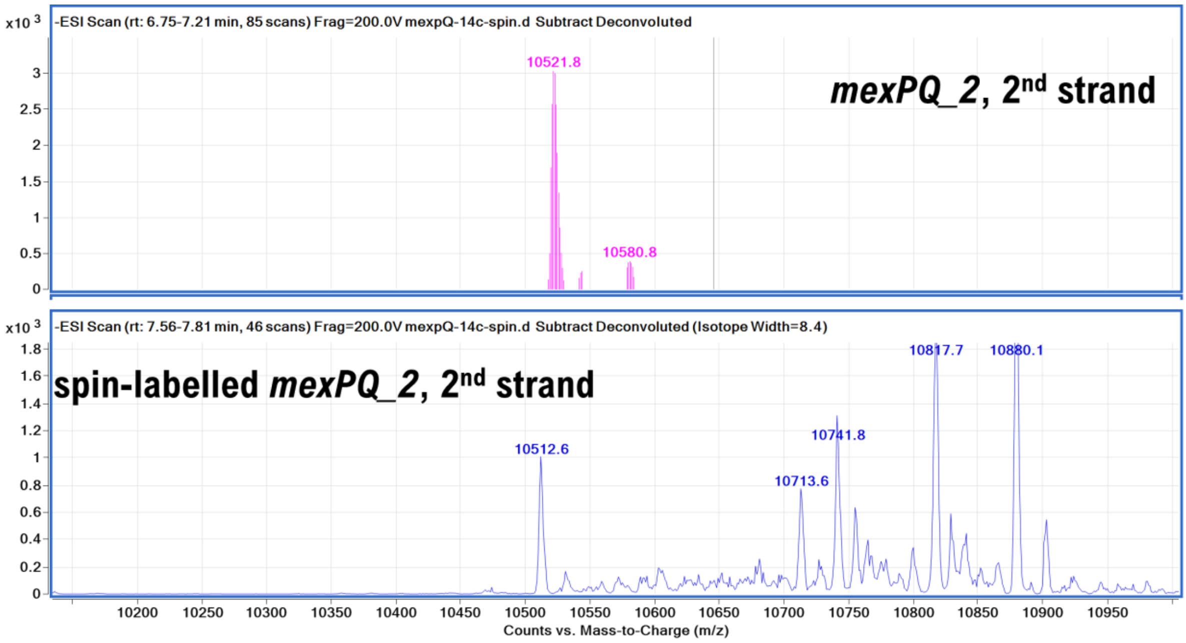

Figure S5: Mass spectrometry data of non-labelled and labelled DNA MexPQ_2.

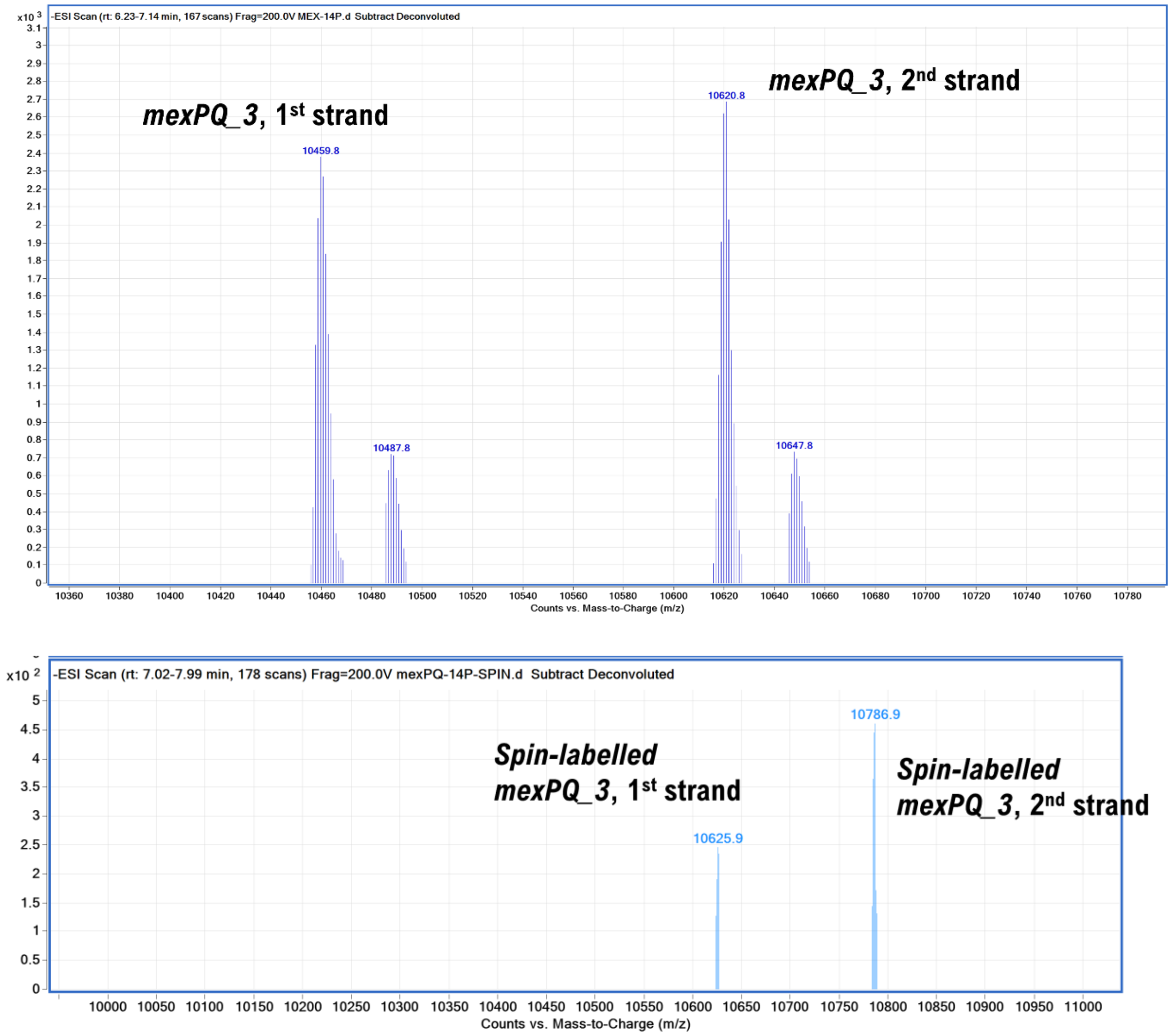

Figure S6: Mass spectrometry data of non-labelled and labelled DNA MexPQ_3.

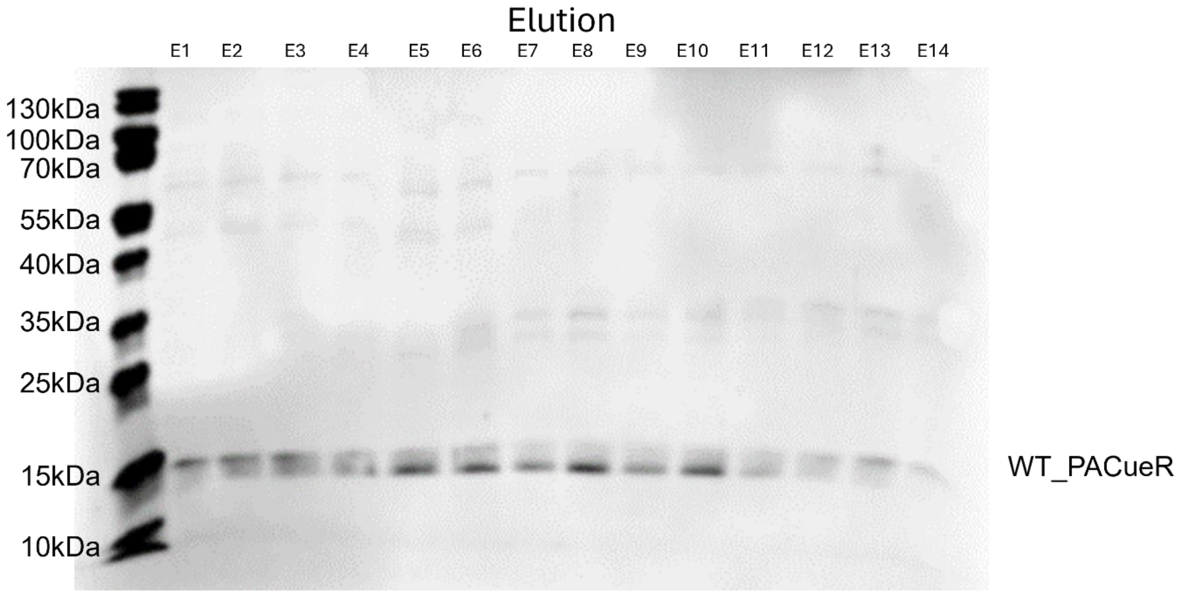

Figure S7: SDS-Gel Picture of purified WT_PACueR.

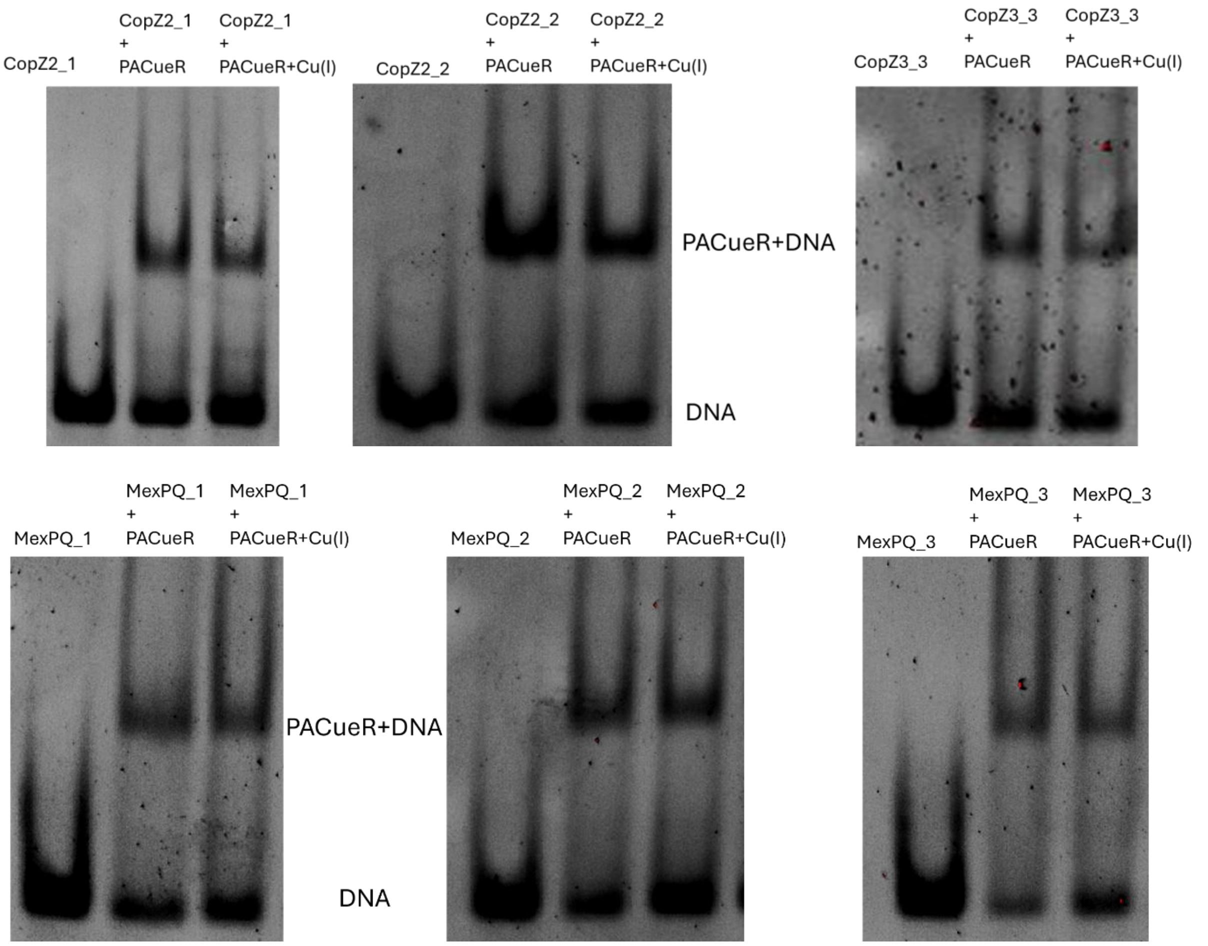

Figure S8: EMSA pictures: complex formation of all spin-labeled DNA promoters used in this study as a function of PACueR and Cu(I) binding.

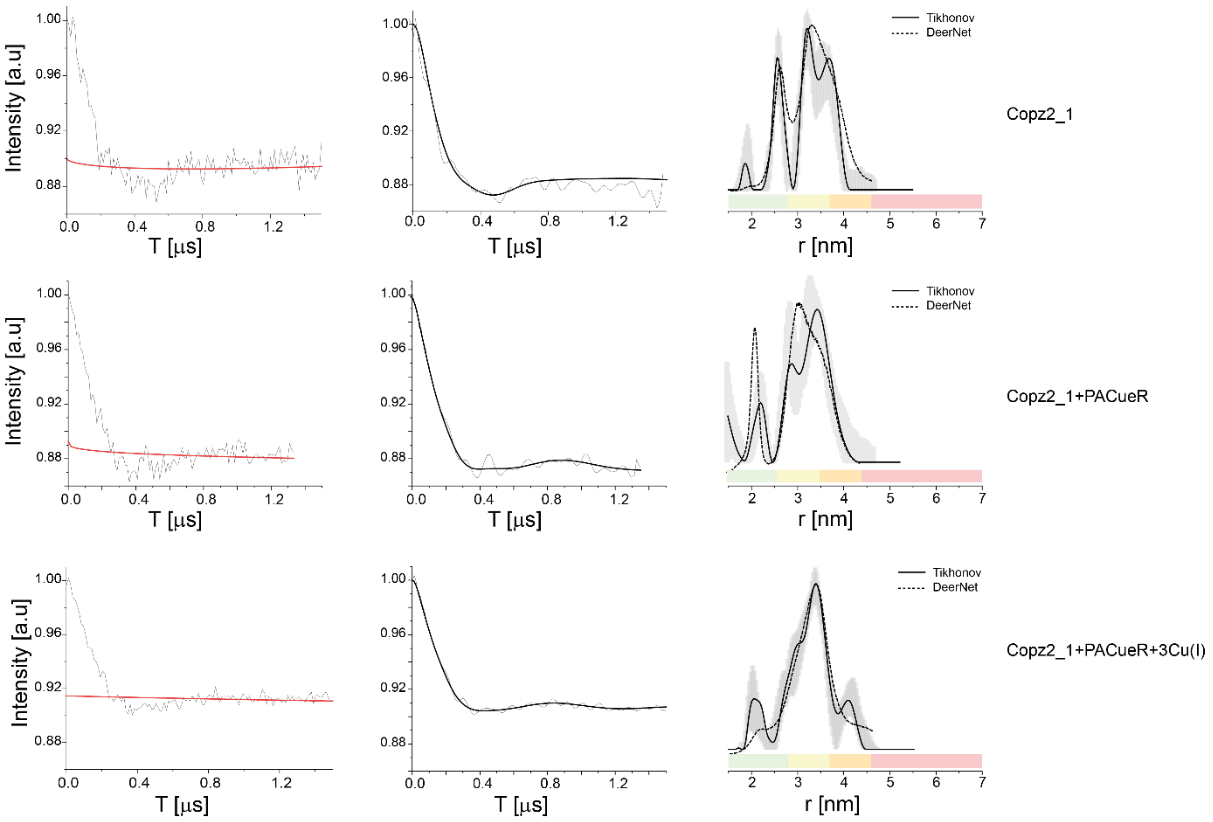

Figure S9: DEER on spin-labelled CopZ2_1 DNA as a function of PACueR and Cu(I) binding: Q-band DEER time domain signals before (left side) and after background subtraction (intermediate) and the corresponding distance distribution function for CopZ2_1 DNA, in the presence of PACueR, and PACueR and Cu(I). The data was analyzed using the DeerAnalysis program using Tikhonov regularization, where the regularization parameter was 20 (solid black lines) and using DEERNet (dashed black lines). Distance distribution validation considered white noise, background start and dimensionality. The colour bar indicates reliability ranges (green: shape reliable; yellow: mean and width reliable; orange: mean reliable; pink: no quantification possible).

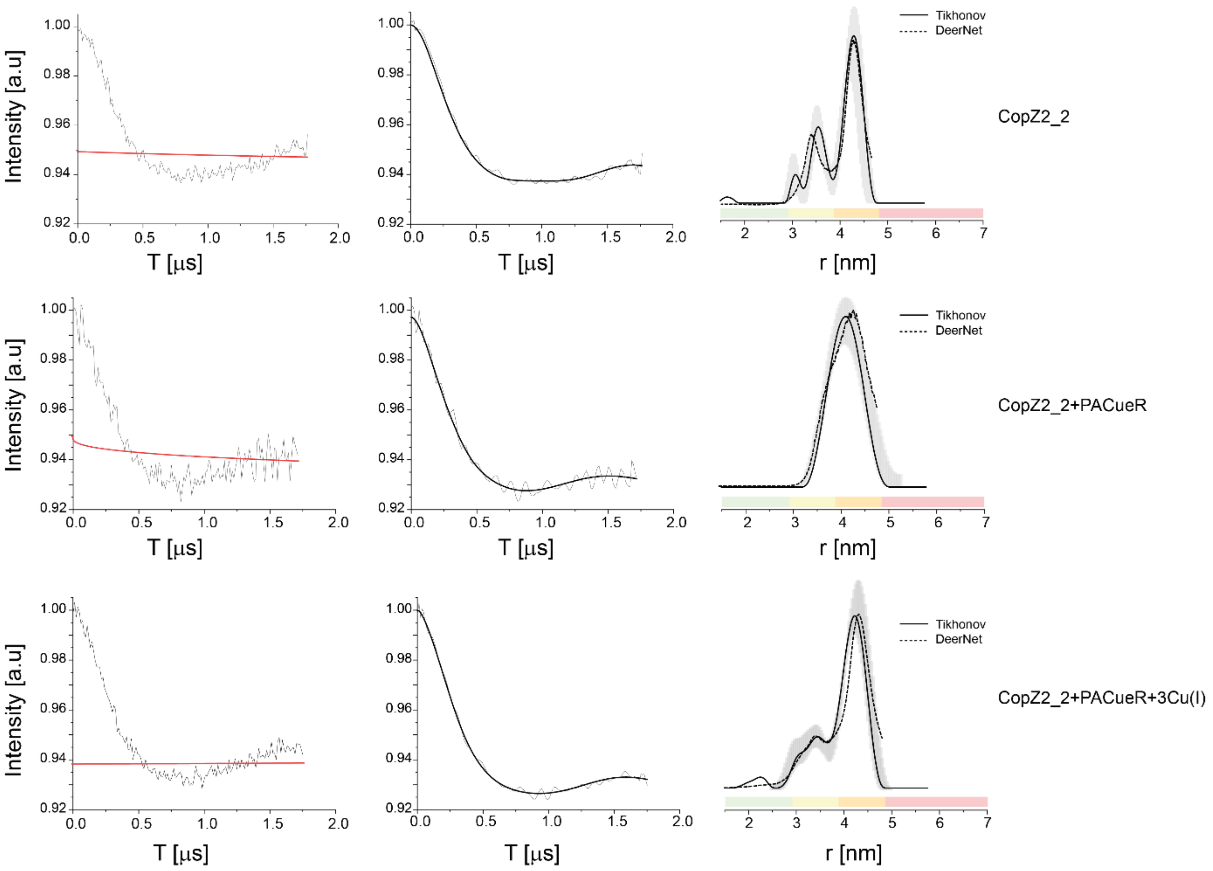

Figure S10: DEER on spin-labelled CopZ2_2 DNA as a function of PACueR and Cu(I) binding: Q-band DEER time domain signals before (left side) and after background subtraction (intermediate) and the corresponding distance distribution function for CopZ2_2 DNA, in the presence of PACueR, and PACueR and Cu(I). The data was analyzed using the DeerAnalysis program using Tikhonov regularization, where the regularization parameter was 20 (solid black lines) and using DEERNet (dashed black lines). Distance distribution validation considered white noise, background start and dimensionality. The colour bar indicates reliability ranges (green: shape reliable; yellow: mean and width reliable; orange: mean reliable; pink: no quantification possible).

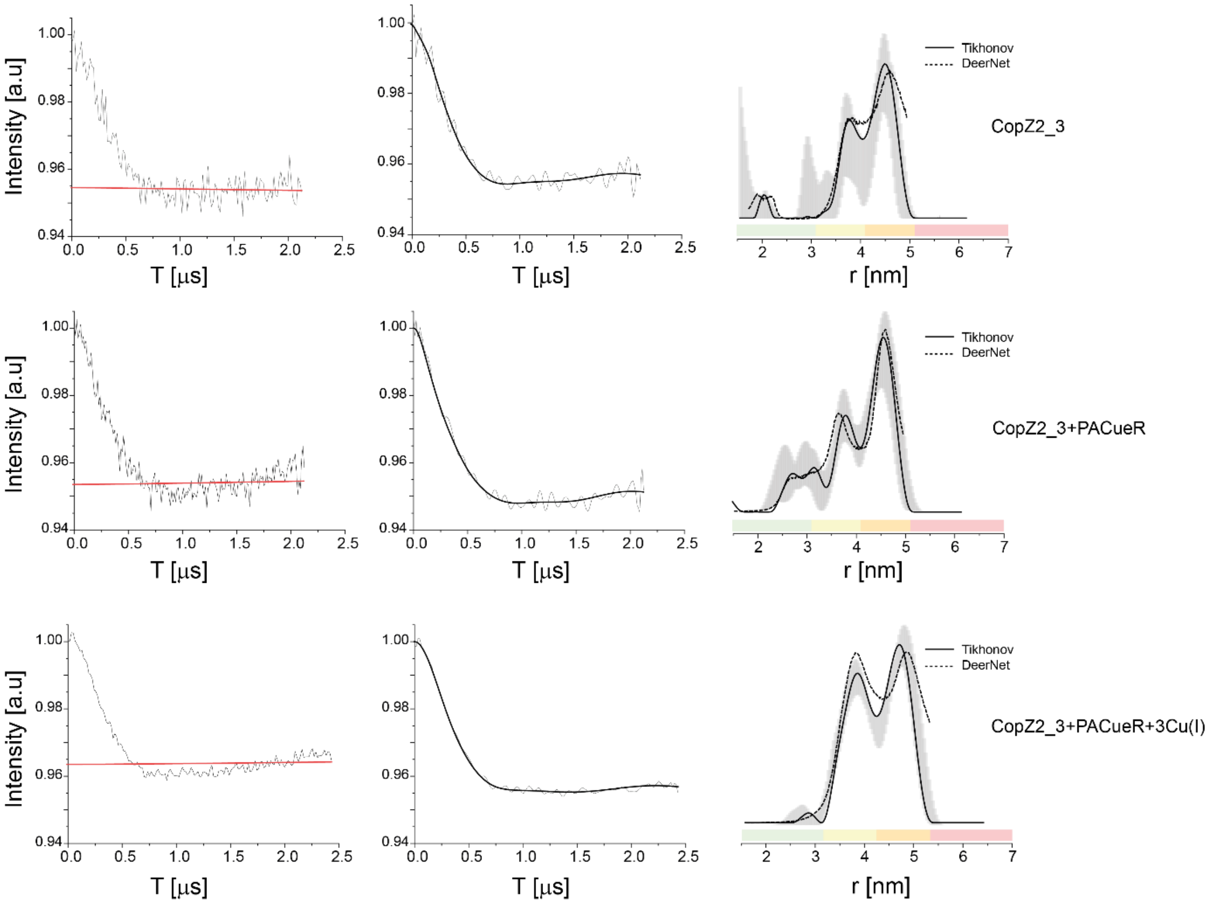

Figure S11: DEER on spin-labelled CopZ2_3 DNA as a function of PACueR and Cu(I) binding: Q-band DEER time domain signals before (left side) and after background subtraction (intermediate) and the corresponding distance distribution function for CopZ2_3 DNA, in the presence of PACueR, and PACueR and Cu(I). The data was analyzed using the DeerAnalysis program using Tikhonov regularization, where the regularization parameter was 20 (solid black lines) and using DEERNet (dashed black lines). Distance distribution validation considered white noise, background start and dimensionality. The colour bar indicates reliability ranges (green: shape reliable; yellow: mean and width reliable; orange: mean reliable; pink: no quantification possible).

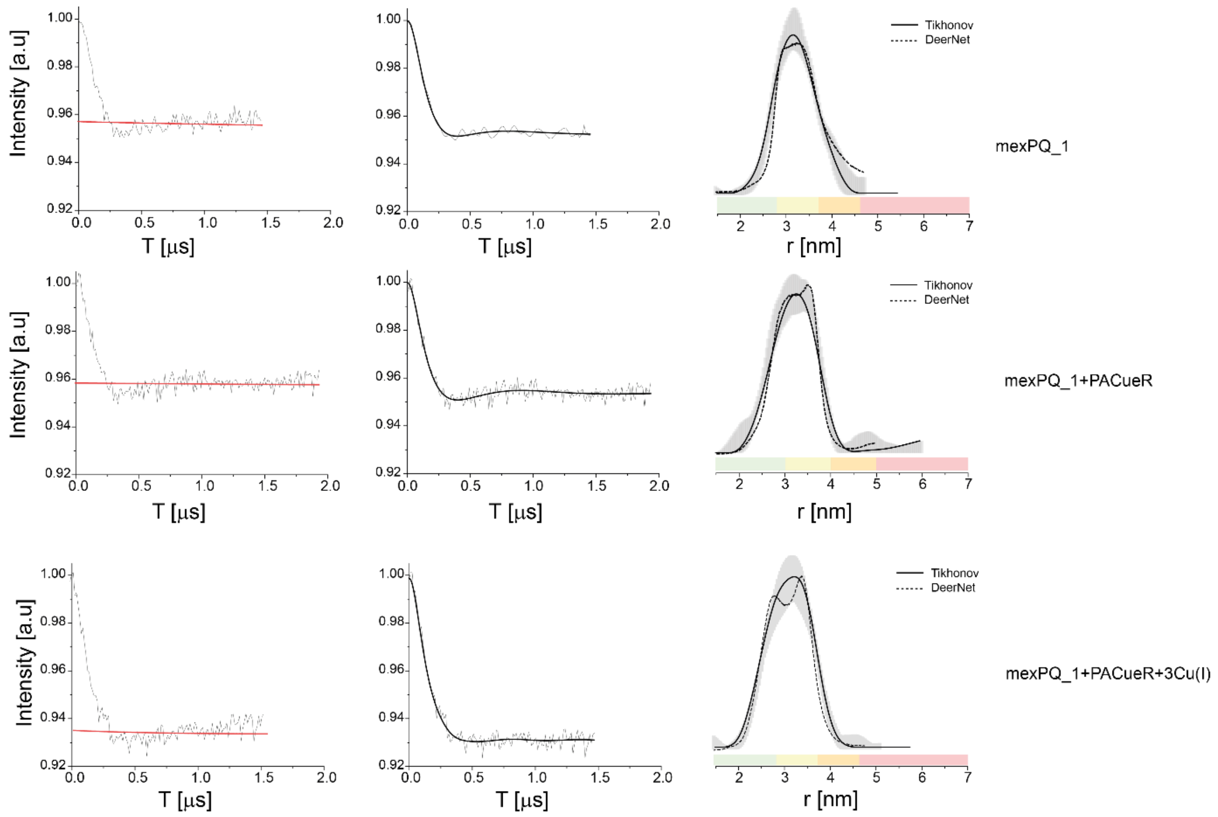

Figure S12: DEER on spin-labelled MexPQ_1 DNA as a function of PACueR and Cu(I) binding: Q-band DEER time domain signals before (left side) and after background subtraction (intermediate) and the corresponding distance distribution function for MexPQ_1 DNA, in the presence of PACueR, and PACueR and Cu(I). The data was analyzed using the DeerAnalysis program using Tikhonov regularization, where the regularization parameter was 20 (solid black lines) and using DEERNet (dashed black lines). Distance distribution validation considered white noise, background start and dimensionality. The colour bar indicates reliability ranges (green: shape reliable; yellow: mean and width reliable; orange: mean reliable; pink: no quantification possible).

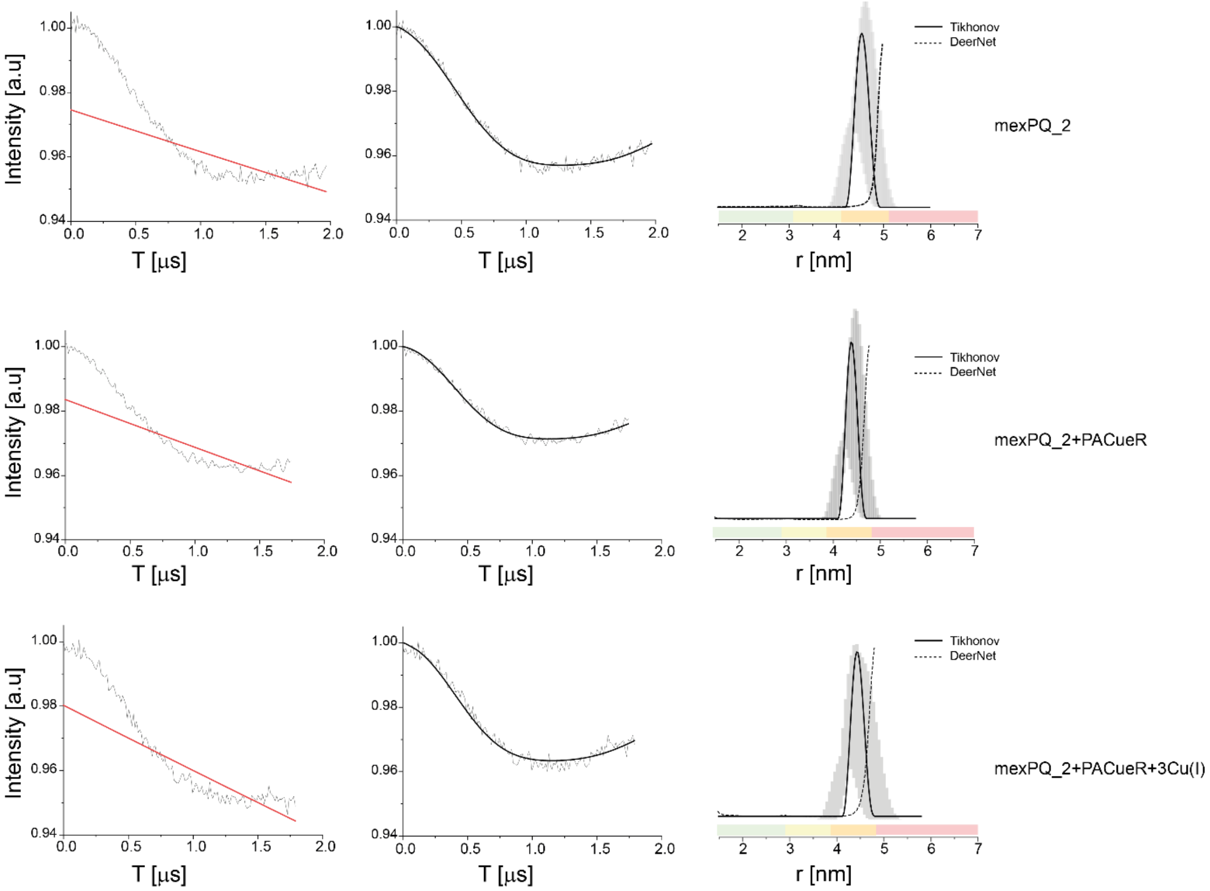

Figure S13: DEER on spin-labelled MexPQ_2 DNA as a function of PACueR and Cu(I) binding: Q-band DEER time domain signals before (left side) and after background subtraction (intermediate) and the corresponding distance distribution function for MexPQ_2 DNA, in the presence of PACueR, and PACueR and Cu(I). The data was analyzed using the DeerAnalysis program using Tikhonov regularization, where the regularization parameter was 20 (solid black lines) and using DEERNet (dashed black lines). Distance distribution validation considered white noise, background start and dimensionality. The colour bar indicates reliability ranges (green: shape reliable; yellow: mean and width reliable; orange: mean reliable; pink: no quantification possible).

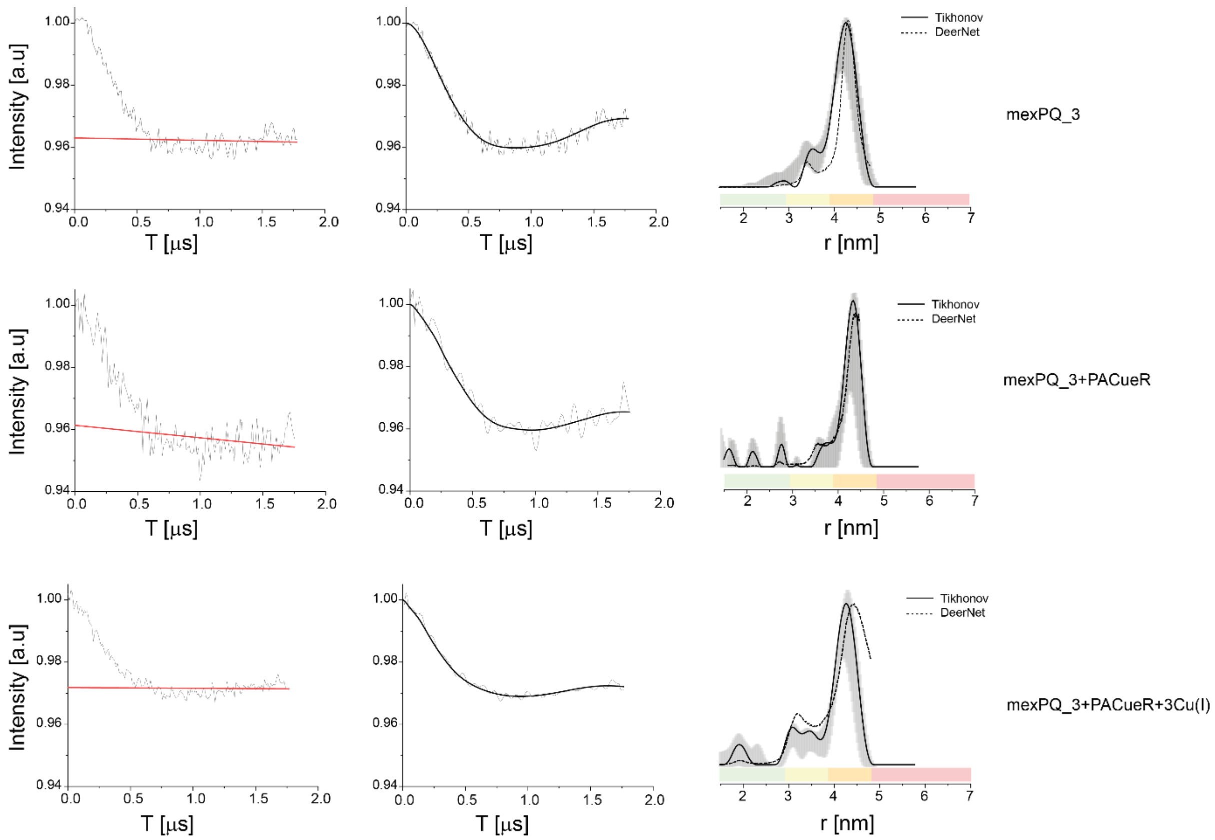

Figure S14: DEER on spin-labelled MexPQ_3 DNA as a function of PACueR and Cu(I) binding: Q-band DEER time domain signals before (left side) and after background subtraction (intermediate) and the corresponding distance distribution function for MexPQ_3 DNA, in the presence of PACueR, and PACueR and Cu(I). The data was analyzed using the DeerAnalysis program using Tikhonov regularization, where the regularization parameter was 20 (solid black lines) and using DEERNet (dashed black lines). Distance distribution validation considered white noise, background start and dimensionality. The colour bar indicates reliability ranges (green: shape reliable; yellow: mean and width reliable; orange: mean reliable; pink: no quantification possible).
